# Prevalence and association of metabolic syndrome and vitamin D deficiency among postmenopausal women in a rural block of West Bengal, India

**DOI:** 10.1371/journal.pone.0188331

**Published:** 2017-11-30

**Authors:** Soumi Srimani, Indranil Saha, Debnath Chaudhuri

**Affiliations:** 1 Department of Biochemistry and Nutrition, All India Institute of Hygiene and Public Health, Kolkata, West Bengal, India; 2 Department of Community Medicine, IQ City Medical College and Narayana Hrudayalaya Hospital, Durgapur, West Bengal, India; University of Alabama at Birmingham, UNITED STATES

## Abstract

**Background:**

Prevalence of metabolic syndrome (MS) and vitamin D deficiency was reported among postmenopausal women (PMW) in India. However, no report is available regarding the association of MS and 25-hydroxyvitamin D [25(OH)D] among PMW in India. This study aimed to find out the prevalence of MS and 25(OH)D status as well as their association among rural PMW of West Bengal, India.

**Materials and methods:**

This cross-sectional study was conducted among 222 randomly selected rural PMW in Singur Block, West Bengal, India. Serum 25(OH)D, Blood pressure (BP), waist circumference (WC), fasting blood glucose (FBG), triglycerides (TG) and high density lipoprotein cholesterol (HDL-C) were measured using standard procedures. MS was defined as per International Diabetes Federation, 2005 (for Asian-Indians) criteria. Statistical tests were done using SPSS software.

**Results:**

Prevalence of metabolic syndrome was 46%. 51% and 19% PMW were vitamin D insufficient and deficient, respectively. 22% and 53% women having MS were vitamin D insufficient and deficient, respectively. Among the PMW, 21% and 47% with WC≥80cm; 22% and 62% with FBG≥110mg/dl; 21% and 54% with TG≥150mg/dl; 23% and 51% with HDL-C<50mg/dl, 15% and 55% with BP≥130/85mm of Hg were vitamin D insufficient and deficient, respectively. Significant statistical association between FBG and 25(OH)D status existed (p = 0.01). Significant positive correlation between WC and 25(OH)D level (p = 0.004) and significant negative correlation between FBG and 25(OH)D level observed (p = 0.02). WC was the only statistically significant predictor of the dependent variable. Odds of non-sufficient 25(OH)D level increased with decrease in WC.

**Conclusion:**

High prevalence of MS as well as vitamin D insufficiency and deficiency existed among PMW of Singur block, West Bengal, India. 25(OH)D had significant inverse and direct relationship with FBG and WC. Low 25(OH)D may be one of the potential risk factors for developing MS in PMW or vice-versa.

## Introduction

Metabolic syndrome (MS), a constellation of the most dangerous heart attack risk factors: diabetes and raised fasting plasma glucose, abdominal obesity, high cholesterol and high blood pressure, is becoming pandemic among the non-communicable diseases (NCD) in today’s world [1–5]. The prevalence of the MS increases with menopause and may partially explain the apparent acceleration in cardio vascular diseases (CVD) after menopause [6]. Prevalence of MS varies in India and different countries [7–12]. Socioeconomic status, increasing urbanization, genetic predisposition, low physical activity, vitamin D deficiency (VDD) and use of high fat, refined sugar and processed foods in diet are the important determinants of MS [13–16]. Low 25-hydroxyvitamin D [25(OH)D] level is associated with obesity, hypertension, diabetes, MS and chronic vascular inflammation, all of which are risk factors for CVD [17–20]. Several studies from many parts of India have established that VDD is widespread among Indians of all age and sex groups including postmenopausal women (PMW), residing in both rural and urban areas [21–24]. Prevalence of vitamin D deficiency and insufficiency among PMW residing in North and South India was reported [25–27]. Only one report is available on the 25(OH)D status among geriatric PMW of West Bengal [28]. However, no community based study report is available regarding the association of MS and 25(OH)Dlevel among the PMW in India.

This study aimed to find out the prevalence of MS and 25(OH)D status as well as their association among rural postmenopausal women of West Bengal, India.

## Subjects & methods

This cross-sectional study was conducted among 222 postmenopausal women, aged 45–70 years, selected randomly from 30 villages of Singur block, the rural field practice area of All India Institute of Hygiene and Public Health (AIIH&PH), West Bengal, India from 27^th^ March, 2014 to 1^st^ August, 2016. Women having history of thyroid dysfunction, on hormonal replacement therapy, amenorrhea due to any pathological cause or surgery, on vitamin D supplementation, physically or mentally challenged and non-cooperative in nature were excluded from the study. Ethical clearance was obtained from the Ethics Committee of AIIH&PH, Kolkata. Informed written consent was obtained prior to the study. Serum 25(OH)D was measured by enzymatic immunoassay [29]. Precision of the estimation was determined by intra assay and inter assay variability. Intra assay (within run variation) was determined by replicate (16x) of the measurement of three different sera in one assay and the variability was ≤ 6.4%. Inter assay (between run variation) was determined by replicate (10x) of three different control sera in different lots of kit and the variability was ≤ 6.95% [29]. VDD, insufficiency and sufficiency was defined as <20 ng/ml, 21–29 ng/ml and 30–100 ng/ml of 25(OH)D in human blood, respectively [30]. Blood pressure (BP), waist circumference (WC), fasting blood glucose (FBG), serum triglycerides (TG) and high density lipoprotein cholesterol (HDL-C) were measured using standard procedures [31–35]. MS was defined as per International Diabetes Federation (IDF), 2005 (for Asian-Indians) criteria [4].

Data were put in Microsoft Excel worksheet (Microsoft, Redwoods, WA, USA) (S1 File) and checked for accuracy. Association between two attributes was calculated by Pearson’s Chi-square test. Continuous data was first checked for normality distribution by Kolmogarov Smirnov Test. Significant P value indicated skewed distribution. Because of skewed distribution, non-parametric tests were performed. Difference between distributions of two continuous variables was determined by Mann Whitney U test (Z value). Correlation was calculated by Spearman’s correlation coefficient (rho). Binary logistic regression was calculated using SPSS software, Version 20.0 (Statistical Package for the Social Sciences Inc, Chicago, IL, USA), by keeping non-sufficient vitamin D level (yes/no) as dependent variable. P value less than 0.05 was considered as statistically significant.

## Results

Out of 222 postmenopausal women, prevalence of MS was found among 46%. 51% and 19% of them were having vitamin D deficiency and insufficiency, respectively.

Out of all the study subjects having MS, 22% and 53% were vitamin D insufficient and deficient, respectively. Among the postmenopausal women, 21% and 47% with WC≥80cm; 22% and 62% with FBG≥110mg/dl; 21% and 54% with TG≥150mg/dl; 23% and 51% with HDL-C<50mg/dl, 15% and 55% with BP≥130/85mm of Hg had vitamin D insufficiency and deficiency, respectively. Significant statistical association was found between FBG with 25(OH)D status using chi-square (χ^2^) test (p = 0.01) (Table 1).

**Table 1:** Distribution of postmenopausal women according to 25(OH)D status in relation with metabolic syndrome, waist circumference, fasting blood glucose, triglyceride, HDL cholesterol and blood pressure (N = 222).

Parameter	25-hydroxyvitamin D status						Total		Statistical test	
	Deficient		Insufficient		Sufficient		*Number*	*Percentage*	*Chi-square statistics*	*p value*
	*Number*	*Percentage*	*Number*	*Percentage*	*Number*	*Percentage*				
****Metabolic syndrome present (IDF)****										
***Yes***	54	53	22	22	26	25	102	100	2.23	0.32
***No***	59	49	20	17	41	34	120	100		
****Waist Circumference****										
***<80cm***	73	47	33	21	51	32	157	100	4.23	0.12
***≥80cm***	40	61	9	14	16	25	65	100		
****Fasting blood glucose****										
***<100mg/dl***	36	62	13	22	9	16	58	100	8.02	0.01
***≥100mg/dl***	77	47	28	18	58	35	164	100		
****Triglyceride****										
***<150mg/dl***	54	54	21	21	25	25	100	100	2.38	0.3
***≥150mg/dl***	59	49	21	17	42	34	122	100		
****HDL cholesterol****										
***<50mg/dl***	40	51	18	23	20	26	78	100	1.92	0.38
***≥50mg/dl***	73	51	24	16	47	33	144	100		
****Blood pressure****										
***≥130/85mm of Hg***	77	55	21	15	42	30	140	100	4.33	0.11
***<130/85mm of Hg***	36	44	21	26	25	30	82	100		

Median 25(OH)D was found to be 20 with a range from 1.4 to 93ng/ml. Median 25(OH)D level varied from 18 to 27 among the PMW having or not having MS. Subjects having MS had significantly lower 25(OH)D level compared to subjects without MS, as revealed by Mann-Whitney U test. A negative correlation was found between 25(OH)Dand MS status; though it was not significant (p = 0.65). Median 25(OH)D level had increased significantly (p = 0.0001) in Mann-Whitney U test) from 16 to 23 in subjects having WC < 80 cm to ≥ 80. There was significant positive correlation between WC and 25(OH)D level (rho = 0.19, p = 0.004). There had been significant negative correlation (rho = -0.15, p = 0.02) between FBG and 25(OH)D level, where median 25(OH)D level significantly decreased (22 to 18) with increase in blood glucose level (<100mg/dl to ≥ 100mg/dl) (p = 0.0001 in Mann-Whitney U test). Median 25(OH)D level decreased (23 to 20) with increase in triglyceride level (<150mg/dl to ≥150mg/dl), while median 25(OH)D level increased (20 to 21) with increase in HDL cholesterol level (< 50mg/dl to ≥ 50mg/dl); and there had been significant difference between these groups in Mann-Whitney U test but no relationship was observed between either of them (p = 0.82 and p = 0.28 respectively) in Spearman test. As per Mann-Whitney U test, median 25(OH)D level significantly decreased from 22 to 19 in both cases with increase in both SBP (<130 mm of Hg to ≥130mm of Hg) and DBP (< 85 mm of Hg to ≥85mm of Hg), respectively but without any significant correlation (p = 0.11 and p = 0.26 respectively) (Table 2).

**Table 2:** Relationship between waist circumference, fasting blood glucose, triglyceride, HDL cholesterol and blood pressure with 25(OH)D level among postmenopausal women (N = 222).

Parameter	25-hydroxyvitamin D		Statistical tests			
	*Median*	*IQR*	Mann Whitney U test		Spearman's correlation test	
			*Z value*	*p value*	*rho*	*p value*
**Metabolic syndrome present (IDF)**						
Yes	18	12–31	-18.23	0.0001	-0.03	0.65
No	27	12–39				
**Waist circumference**						
<80cm	16	10–29	-16.58	0.0001	0.19	0.004
≥80cm	23	14–37				
**Fasting blood glucose**						
<100mg/dl	22	13–38	-16.06	0.0001	-0.15	0.02
≥100mg/dl	18	11–27				
**Triglyceride**						
<150mg/dl	23	11–37	-17.89	0.0001	0.15	0.82
≥150mg/dl	20	13–30				
**HDL cholesterol**						
<50mg/dl	20	12–30	-14.66	0.0001	0.07	0.28
≥50mg/dl	21	12–39				
**Blood pressure**						
SBP<130mm of Hg	22	13–31	-18.23	0.0001	-0.1	0.11
SBP≥130mm of Hg	19	12–34				
DBP<85mm of Hg	22	13–31	-17.54	0.0001	-0.07	0.26
DBP≥85mm of Hg	19	12–37				

The binary logistic regression model is significant as evident from significant Omnibus Chi-square statistic (P = 0.01) and non-significant Hosmer-Lemeshow statistics (0.20). Independent variables can explain 7%– 9.9% variance of dependent variable from Cox & Snell R square and Nagelkerke R square values. The model correctly predicted 4.5% of sufficient 25(OH)D level and 94.8% of non-sufficient 25(OH)D level from classification table. Only WC became the significant predictor of the dependent variable. Odds of non-sufficient 25(OH)D level increased with decrease in WC (Table 3).

**Table 3:** Binary logistic multivariable regression model analysis of 25(OH)D level.

Variables in the Equation									
Parameter		B	S. E.	Wald	df	Sig.	Exp (B)	95% C. I. for Exp(B)	
								*Lower*	*Upper*
**Step 1**^a^	***SBP***	0.02	0.01	0.61	1.00	0.43	1.01	0.99	1.03
	***DBP***	0.000	0.02	0.000	1.00	0.98	1.00	0.96	1.04
	***HDL***	0.007	0.01	0.44	1.00	0.51	0.99	0.97	1.01
	***TG***	0.004	0.002	3.01	1.00	0.08	1.004	1.00	1.008
	***WC***	0.03	0.01	5.77	1.00	0.02	0.97	0.94	0.99
	***FBS***	0.008	0.005	2.83	1.00	0.09	1.01	0.99	1.02
	***Constant***	1.49	1.54	0.93	1.00	0.34	4.42		

^a^Variable(s) entered on Step 1: SBP, DBP, HDL, TG, WC, FBS.

## Discussion

Our study revealed that almost half (46%) of the PMW under investigation were having MS. Prevalence of MS varies in India and different countries; 55% in urban Western India, 64.3%in Iran, 49.8% in Brazil, 16.9% in Thailand and 29% in Puerto Rico [7, 10, 11, 36, 37]. We have found that among 51% and 19% PMW, in the study area, were suffering from vitamin D deficiency and insufficiency, respectively. VDD was thought to be rare in India due to their exposure to sunshine which is supported by the results of earlier epidemiological studies of the frequencies of rickets and osteomalacia in Indian sub-continent [38]. Earlier studies reported that 70% and 23% of South Indian and 53.35% and 19.48% of North Indian PMW had <20ng/ml and 21-29ng/ml of 25(OH)D respectively [25, 39]. Only one study from West Bengal among 65 geriatric PMW reported that 37, 9 and 19 women had deficient, insufficient and normal serum 25(OH)D3 level [28]. Data from other studies on PMW revealed that, 42% and 92% of Brazilian women [40] and South Korean women [41] had <30 ng/mL of 25-hydroxy cholecalciferol, respectively. Severe deficiency (≤10 ng/mL) was most prevalent in South Asia and the Middle East [42]. Earlier studies therefore revealed existence of country wise variations of both MS and serum 25(OH)D level. Considering deficiency of vitamin D as one of the factors for developing MS, it is pertinent to determine the association between MS as well as its components and serum 25(OH)D level.

We have found significant difference in median 25(OH)D level between the groups (normal and abnormal) of each parameter of MS including itself. Significant association between 25(OH)D and FBG was observed. However, no significant association between 25(OH)Dstatus with MS and its other parameters was observed. FBG was inversely correlated with serum 25(OH)D level and this relation was found to be statistically significant. Though, inverse relationship between 25(OH)D level with MS, SBP and DBP was observed, it was not statistically significant. Significant positive correlation was observed between 25(OH)D and WC; however, the relationship with TG and HDL-C, though positive, but non-significant.

Various epidemiological studies have shown that patients with type 2 diabetes mellitus (T2DM), one of the components of MS, had significantly lower circulating concentrations of 25(OH)D, compared to healthy controls [43–46].Studies conducted by Pittas, et al. in 2007 and Chowdhury, et al. in 2009 concluded that low levels of 25(OH)D may negatively influence glycaemia [47,48]. Several other epidemiological studies, conducted in different countries reported that low circulating vitamin D concentration may be associated with an increased prevalence of hyperglycaemia, MS, WC, serum TG [20, 44–46, 48–56] and increased risk for cardiac events [56–60]. In a meta-analysis of ten observational studies and nine randomised control trials, associations between 25(OH)D levels and BP was found [60]. Eight observational studies and three randomised control trials supported an inverse association between vitamin D and BP [60]. All these reports as well as findings from the present study indicate that the cause of MS and the abnormalities of its components was due to low circulating 25(OH)D but it will be of interest to explore whether MS and its components, particularly WC, are responsible for lowering circulating 25(OH)D or not.

In recent times, a previously unrecognized alternative pathway of vitamin D activation, initiated by C20-hydroxylation of vitamin D by CYP11A1, has been confirmed to operate *in vivo*, generating novel D3-hydroxyderivatives different from 25(OH)D and 1,25(OH)_2_D [61,62]. Slominski AT et. al. detected the predominant metabolite 20(OH)D of this novel pathway in human serum with a relative concentration ∼20 times lower than 25(OH)D [61]. Further studies detected CYP11A1-derived secosteroids, including 20(OH)D, of this alternative pathway, in the human serum and epidermis whose biological activity suggest that they act as hormones *in vivo* [63]. Therefore, future studies may explore to find out the possible relationship of MS with 20(OH)D and other secosteriods of this alternative pathway.

Thus, it can be concluded that, high prevalence of metabolic syndrome as well as vitamin D deficiency or insufficiency existed among postmenopausal women of Singur block, West Bengal, India. 25(OH)D level has significant inverse relationship with blood glucose level and direct relationship with waist circumference. Vitamin D deficiency or insufficiency, therefore, may be one of the potential risk factors for developing MS in the studied population or vice versa.

## Supporting information

S1 File Data sheet. (XLSX)
